# Studies on the Powerful Photoluminescence of the Lu_2_O_3_:Eu^3+^ System in the Form of Ceramic Powders and Crystallized Aerogels

**DOI:** 10.3390/gels10110736

**Published:** 2024-11-13

**Authors:** Alan D. Alcantar Mendoza, Antonieta García Murillo, Felipe de J. Carrillo Romo, José Guzmán Mendoza

**Affiliations:** 1 Instituto Politécnico Nacional CIITEC, Azcapotzalco, Mexico City 02250, Mexico; a_daniel_am@outlook.com (A.D.A.M.); fcarrillo@ipn.mx (F.d.J.C.R.); 2 Instituto Politécnico Nacional CICATA, Legaria, Mexico City 11500, Mexico; joguzman@ipn.mx

**Keywords:** rare earths, aerogel, photoluminescence

## Abstract

This study compared the chemical, structural, and luminescent properties of xerogel-based ceramic powders (CPs) with those of a new series of crystallized aerogels (CAs) synthesized by the epoxy-assisted sol–gel process. Materials with different proportions of Eu^3+^ (2, 5, 8, and 10 mol%) were synthesized in Lu_2_O_3_ host matrices, as well as a Eu_2_O_3_ matrix for comparative purposes. The products were analyzed by infrared spectroscopy (IR), X-ray diffraction (XRD), scanning electron microscopy (SEM) with energy-dispersive spectroscopy (EDS), transmission electron microscopy (TEM), photoluminescence analysis, and by the Brunauer–Emmett–Teller (BET) technique. The results show a band associated with the M-O bond, located at around 575 cm^−1^. XRD enabled us to check two ensembles: matrices (Lu_2_O_3_ or Eu_2_O_3_) and doping (Lu_2_O_3_:Eu^3+^) with appropriate chemical compositions featuring C-type crystal structures and intense reflections by the (222) plane, with an interplanar distance of around 0.3 nm. Also, the porous morphology presented by the materials consisted of interconnected particles that formed three-dimensional networks. Finally, emission bands due to the energy transitions (^5^D_J_, where J = 0, 1, 2, and 3) were caused by the Eu^3+^ ions. The samples doped at 10 mol% showed orange-pink photoluminescence and had the longest disintegration times and greatest quantum yields with respect to the crystallized Eu_2_O_3_ aerogel.

## 1. Introduction

Materials synthesized from rare-earth (RE) elements, commonly used in the form of oxides (REOs), have a wide diversity of applications directly related to their properties. Among these, they have become indispensable due to their fascinating and highly efficient luminescent properties, which arise from transitions of the f orbitals that are shielded by the external s and p orbitals [1,2,3,4]. That is why they can be found widely applied as phosphors used for lighting in ceramics, display screens, security label inks, lasers, fiber optics, night vision devices, biomarkers, nanothermometers, nanoscopy, and the administration of activated drugs [5,6,7,8,9,10,11]. Several well-known, commercial phosphors have been used to meet the needs of these applications; some of the most popular are Y_2_O_3_:Eu^3+^, Y_2_O_2_S:Eu^3+^, BaMgAl_10_O_17_:Eu^2+^, and MgAl_11_O_19_:Ce^3+^ [12]. Commonly, REO phosphors are synthesized by chemical methods such as hydrothermal, capping, cluster-generation, and electrochemical methods [2,13].

However, authors such as Tshikovi et al. [14] have indicated that the sol–gel process is the most effective method for the preparation of luminescent materials as they have impurities in their structuring. Among the rare-earth trivalent ions (RE^3+^), Eu^3+^ and Tb^3+^ ions are commonly used as luminescence-activating dopants. Moreover, the sol–gel process has established itself as an extremely effective method because of its advantages such as synthesis at lower temperatures and the high homogeneity and purity that can be achieved in processing high-end materials [15,16]. In addition, the epoxy-assisted variant has been widely used in recent years to synthesize the gels of metal oxide compositions; among other benefits, it can be doped with RE ions to modify their properties or induce new ones. In this sense, several studies using this variant can be found, involving the production of porous materials such as REO xerogels or REO aerogels [8,9,17,18,19,20], although it should be mentioned that in these works, the luminescent properties of the materials were marginal or completely disregarded.

Furthermore, aerogels are a type of material that, despite having been discovered more than 90 years ago by Samuel Kistler [21], have not yet been fully exploited [22] due to their high production cost, which is why they are researched and synthesized only in very small quantities in laboratories [23,24]. These materials are made up of a three-dimensional network of interconnected particles, which is extremely fragile due to the huge number of “chains” of pores (micro- and mesopores) that it possesses [25,26], although this same porosity is the basis of its physicochemical properties [27]. With the combination of a macroscopic external structure and a highly specific surface area on the nanoscale, these materials have seen greatly increased use in novel technologies in different areas [28], for example, in aerospace technology [29], construction [30], and energy storage [17]. More recently, their potential use in nano biomedicine has been investigated for its application as a drug carrier [22,24,27,31]. Overall, aerogels are very interesting materials but complicated to manipulate and even more difficult to characterize.

As mentioned, the common way to obtain aerogels is through the sol–gel process [32,33,34,35], where, once the gel is obtained, it must be carefully treated to extract all the liquid phases inside. What makes the difference in the porosity of aerogels and xerogels and the pattern of particle dispersion is the type of drying [24,36,37]. Xerogels are made up of a series of “collapsed” particles in the three-dimensional network of interconnected particles that form the gel. When these materials are dried at room temperature and sintered, the result is an agglomeration of material that can be defined as ceramic powders. To prevent the three-dimensional network from collapsing due to surface tension and capillary forces during drying, supercritical CO_2_ (scCO_2_) drying is used as the most effective technique for replacing the liquid phase of the gels with a gaseous phase (which, in this case, is air). As a consequence, scCO_2_ causes only a slight contraction of the original three-dimensional structure of the gel, keeping the three-dimensional network of interconnected particles intact, or “open” [23,36,38,39,40]; scCO_2_ is especially effective and is applied in the production of aerogels to obtain better stability and durability [26,27].

In general, especially when it comes to aerogels, there is a limited amount of work dedicated to the investigation of porous lutetium sesquioxide (Lu_2_O_3_) materials. However, there is a large amount of research related to photoluminescent materials based on Lu_2_O_3_:RE^3+^ systems [41,42,43,44,45,46,47]. Some of the most notable works in this area are reviewed below.

Jia et al. [48] reported their synthesis of luminescent Lu_2_O_3_:Ln^3+^ (Ln = Eu, Er, Yb) microspheres using a precipitation method. After being calcined at 800 °C for 2 h, Ln^3+^-doped products were able to index into the cubic Lu_2_O_3_ phase. The sample Lu_2_O_3_:5%Eu^3+^ especially produced strong red emissions caused by the excitation of the charge transfer band (CTB) at approximately 245 nm and was subsequently found to be dominated by the ^5^D_0_-^7^F_2_ transition of Eu^3+^ ions. The decay curve indicated that the lifetime of the Eu^3+^ ions was 1.21 ms. Similarly, Gao et al. [49] synthesized microspheres using a precipitation method; these were calcined at different temperatures, and it was determined that at 800 °C, cubic phases were obtained with well-defined diffraction peaks, with a d222 distance of 0.300 nm. The abundant luminescence emitted by the microspheres was dominated by the “red” ^5^D_0_-^7^F_2_ transition (613 nm), which is described as an electric-dipole-allowed transition that is hypersensitive to the environment. Also, Zhao et al. [4] managed to synthesize “3D hierarchical architectures” of europium-doped lutetium oxide using hydrothermal and calcination processes. The phosphors exhibited polycrystalline cubic-type structures, with the strongest red emissions caused by transitions ^5^D_0_ → ^7^F_J_ (J = 0, 1, 2, 3, and 4) with lifetimes of 1.23, 1.49, and 1.19 ms. Later, in a similar study, Popielarski et al. [50] synthesized ball-like Lu_2_O_3_:Eu^3+^ nanoparticles using the hydrothermal process, which, if carefully observed, could be considered to have a coralliferous morphology. These materials emitted photoluminescence due to the ^5^D_0_ → ^7^D_J_ transitions of the Eu^3+^ ions at the C2 sites. The decay time when doping the Lu_2_O_3_:3%Eu was 1.5 ms. More recently, Tang et al. [51], using a two-step precipitation method, synthesized dispersed spherical particles that were annealed at 800 °C for 2 h, resulting in cubic structures associated with the space group Ia3. The Lu_2_O_3_:Eu^3+^ sample showed luminescence due to Eu^3+^ ion transitions, and the 5 mol% Eu^3+^ sample had a decay time of 2.04 ms. Finally, Olvera Salazar et al. [16] conducted one of the few experiments where a wide series of Lu_2_O_3_ xerogels were synthesized at different doping concentrations of Eu^3+^. In addition, nanoparticles synthesized by the sol–gel method and heat-treated up to 700 °C for one hour are described as novel and potential antioxidant materials, although, unfortunately, the luminescent properties of these materials were not studied.

There are some commonalities in the findings of these studies, for example, Lu_2_O_3_ was found to be an excellent candidate as a host matrix for luminescent lanthanide ions due to its favorable physical properties such as a high melting point, phase stability, and low thermal expansion, among others. Moreover, materials based on the Lu_2_O_3_:TR^3+^ system have extremely attractive optical characteristics stemming from 4f-5d electronic transitions, which leads to their recommendation for applications in areas such as advanced flat displays, high-security technologies, and biological labeling, due to their excellent dispersion and luminescence properties. Studying or proposing new synthesis methods and materials for the Lu_2_O_3_:TR^3+^ system, such as the epoxide-assisted sol–gel method of obtaining aerogels, can reduce the production costs of the system and yield more efficient materials that can compete with current commercial products.

## 2. Results and Discussion

In this work, through the epoxy-assisted sol–gel process, ceramic powders (CPs) were synthesized from xerogels and crystallized aerogels (CAs) using different proportions of Eu^3+^ ions (2, 5, 8, and 10 mol%) in Lu_2_O_3_ host matrices, as well as in a europium sesquioxide (Eu_2_O_3_) matrix, to enable their chemical, structural, and photoluminescent properties to be compared. The labels and the general characteristics of the synthesized samples are shown in Table 1.

### 2.1. Infrared (IR) Spectroscopy

**Figure 1:**
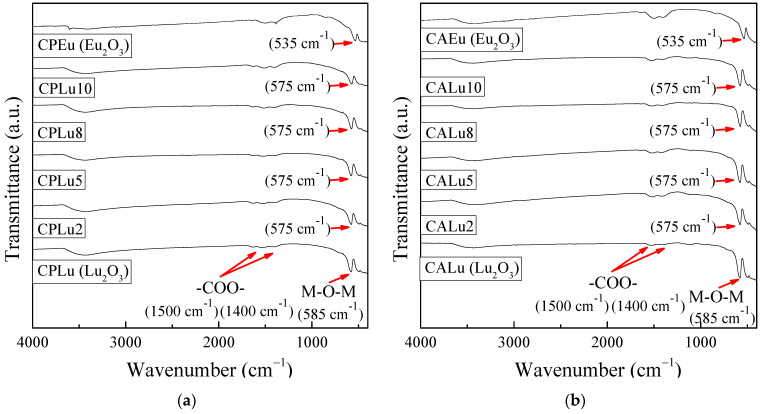
IR spectra of Lu_2_O_3_:Eu^3+^: (**a**) ceramic powders and (**b**) crystallized aerogels.

Figure 1 presents the IR spectra of the CPs and CAs. The results show low signals corresponding to the -COO- interaction around 1500 and 1400 cm^−1^. This interaction is mainly attributable to atmospheric remains; however, the interaction was not constant and was more noticeable in the CPEu and CAEu samples. The main stretching vibration M-O band, which is associated with the interactions of the REs in the formation of REOs, was detected at a position of 585 cm^−1^ for the Lu_2_O_3_ host sample and at 535 cm^−1^ for the Eu_2_O_3_ matrix sample [49], while for all the doped samples, both the CPs and CAs, it was located at 575 cm^−1^. This change occurred due to the incorporation of Eu^3+^ in the doped samples.

This has not been reported in other investigations such as [4,51,52]; they mention that the band was located around 570 cm^−1^, but it was attributed only to the stretching vibrations of lutetium. Also, it can be supposed that if the concentration of Eu_2_O_3_ was increased in the Lu_2_O_3_ host matrix, this band would show a greater displacement toward the Eu_2_O_3_ position (535 cm^−1^).

### 2.2. X-Ray Diffraction (XRD)

**Figure 2:**
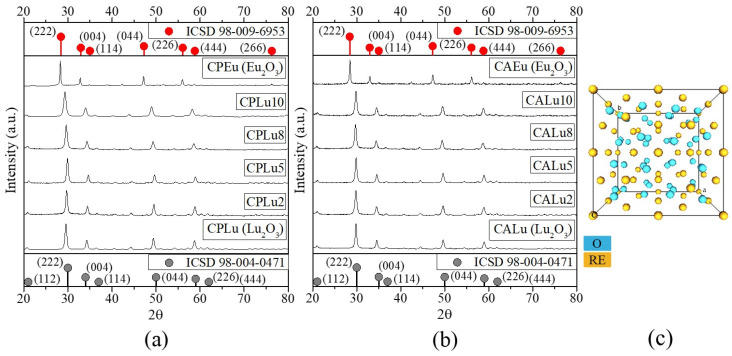
XRD patterns of the (**a**) ceramic powders, (**b**) crystallized aerogels of the Lu_2_O_3_:Eu^3+^ system and (**c**) schematic representation of the crystalline structure of C-type RE_2_O_3_.

The XRD patterns of both the CP and CA materials are shown in Figure 2a,b, where a series of peaks can be seen that correspond with the diffraction charts ICSD 98-004-0471 and ICSD 98-009-6953, for Lu_2_O_3_ and Eu_2_O_3_, respectively. The plane with the highest intensity (222) indicates high purity and well-defined crystalline structures of type C with an Ia-3 space group, like the one presented in Figure 2c. This is very important for REO luminescent materials because high crystallinity generally means fewer traps and stronger luminescence [48]. Furthermore, unlike what was seen in [8] where rare-earth aerogels were synthesized with a similar methodology, in our work, no secondary phases caused by Cl^−^ ions appeared. In particular, the planes suffered a slight displacement in relation to the Lu_2_O_3_ host matrix due to the incorporation of the Eu^3+^ ions that replaced Lu^3+^ [49].

As in the case of IR spectroscopy, if the concentration were to be increased further, the peaks would tend more toward the peaks in the Eu_2_O_3_ matrix. This behavior is due to the incorporation of the europium atoms, which causes deformations in the crystalline structure and results in different average sizes of the crystallites. These effects were calculated with the Debye–Scherrer equation and are presented in Table 2.

Although the sizes varied, there was a tendency to form new crystals on the part of both the CAs and the CPs. This occurred because, at a concentration of 2 mol%, the Lu_2_O_3_ crystallites tended to be smaller; at the next concentration (5 mol%) they reached an average size similar to that of the Lu_2_O_3_ host. When the Lu_2_O_3_ was ±17 nm, they formed with dimensions less than 10 nm (CPLu8 = 9.6 nm and CALu8 = 8.2 nm) to reach their final size in the samples doped at 10 mol%. It was not possible to calculate the average crystallite size for the Eu_2_O_3_ matrix. The results of the Rietveld analysis are presented in Table 2, which shows that the interplanar distance from the (222) plane was around 0.3 nm for all the samples.

**Figure 3:**
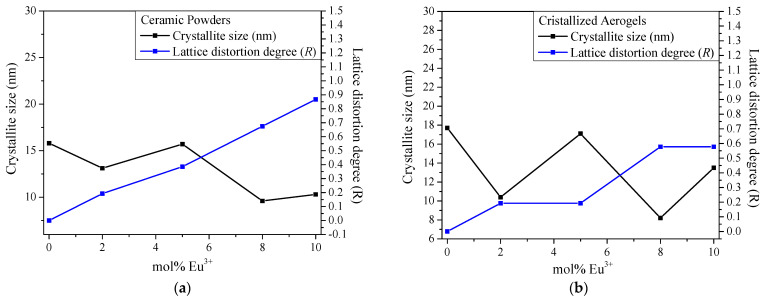
Crystallite size vs. lattice distortion degree for (**a**) ceramic powders and (**b**) crystallized aerogels.

The smaller crystallite size for the doped samples at 8 mol% could be related to this doping concentration via two possible effects: (1) reduced crystal growth rates and (2) enhanced nucleation sites. With reduced crystal growth rates, europium ions create lattice distortions in the Lu_2_O_3_ host, limiting crystal growth and forming larger grains. Similar behavior was reported by Nair et al. [53] and Shakirzyanov et al. [54], where the lattice distortion degree *R* allowed the quantification of the deviation of Lu-O bond lengths from their ideal values in the undoped structure. The doping-induced effect on REO bond length (L) and the lattice distortion degree (*R*) parameter were estimated using relations (1) and (2), respectively [55]:

(1) $$L=\frac{a\sqrt{3}}{4}$$

(2) $$R=\frac{\left|L_{\mathrm{doped}}-L_{\mathrm{undoped}}\right|}{L_{\mathrm{doped}}}\;\times \;100$$

where a is the lattice parameters obtained from XRD, $L_{\mathrm{doped}}$ is the Lu-O bond length in the doped structure, and $L_{\mathrm{undoped}}$ is the bond length in undoped Lu-O. Figure 3a,b shows the relationship between crystal size (nm) and lattice distortion degree (R) as a function of mol% Eu^3+^ for ceramic powders and crystallized aerogels.

When Lu^3+^ is substituted with Eu^3+^, a lattice distortion degree is observed that increases with the Eu^3+^ molar concentration, i.e., ranging from 0.67 to 0.57 for CPLu8 and CALu8. This is due to the different ion radii between Lu^3+^ and Eu^3+^. In both samples at 8 mol%, we can observe the atomic-scale distortions that lead to the formation of the smallest crystallite sizes observed for the two systems.

Lanthanide contraction refers to an unexpected decrease in the atomic and ionic radii of this family of elements [56]. It is caused by poor screening of the 6 s electrons from nuclear displacement by the 4f electrons, which results in smaller atoms (from lanthanum to lutetium) due to an increase in the effective nuclear charge to attract each electron. The unit cell parameters of the samples are presented in Table 3. These confirm the successful production of cubic crystal structures (Ia-3), and although the samples have different values, the behavior induced by the lanthanide contraction is interesting. The cell volume would be expected to increase as the amount of Eu^3+^ doping increases, which happens inversely because the size of the europium atom is larger than the lutetium atom.

### 2.3. Scanning Electron Microscopy (SEM)

**Figure 4:**
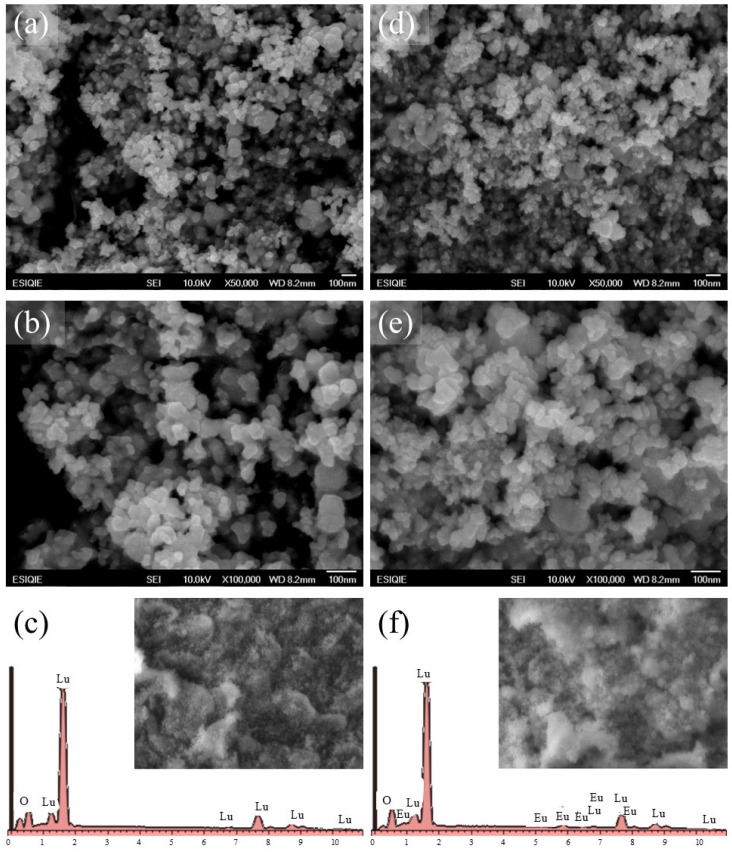
SEM images at different magnifications of (**a**) ×50,000 and (**b**) ×100,000, and (**c**) EDS analysis of the CPLu sample. SEM images at different magnifications of (**d**) ×50,000 and (**e**) ×100,000, and (**f**) EDS analysis of the CPLu10 sample.

Figure 4 shows micrographs of the CP samples CPLu (a and b) and CPLu10 (d and e). These exhibit a “collapsed” three-dimensional network made up of many agglomerated particles, with irregular shapes and sizes of less than 100 nm. The results of the elemental analysis by EDS for the host matrix sample CPLu (Figure 4c) only show the signals corresponding to the compound Lu_2_O_3_, while for the doped sample CPLu10 (Figure 4f), the signals associated with the presence of europium are also seen.

**Figure 5:**
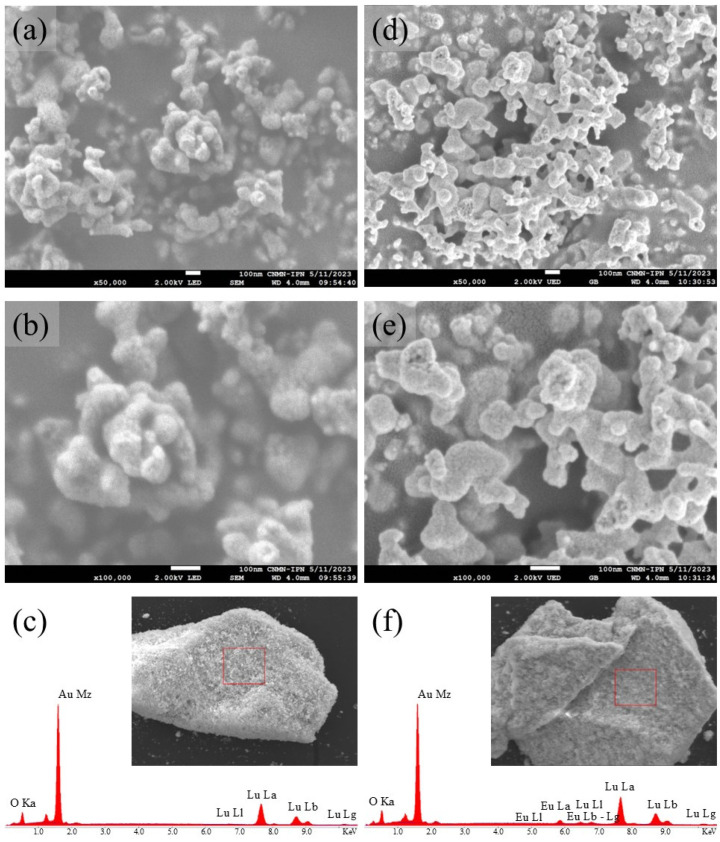
SEM images at different magnifications of (**a**) ×50,000 and (**b**) ×100,000, and (**c**) EDS analysis of the CALu sample. SEM images at different magnifications of (**d**) ×50,000 and (**e**) ×100,000, and (**f**) EDS analysis of the CALu10 sample.

Naturally, the CAs (Figure 5) presented morphological differences. Although many particles with irregular shapes continued to appear, in this case, the microstructure is formed by an “open” interconnection of particles forming three-dimensional networks. In other work, these have been described as coral-shaped structures [18]. This type of particle dispersion is directly related to high porosity [38,57], specifically, the high porosity provided by the chains of pores that form between the particles due to the adequate extraction of CH_3_CH_2_OH during scCO_2_, but in the case of the Lu_2_O_3_:Eu^3+^ system, this type of morphology is reported here for the first time.

For the elemental analysis of the CALu and CALu10 aerogels (Figure 5c,f), the same congruence is maintained as in the CPs. Thus confirming the possibility of including Eu^3+^ in the composition of Lu_2_O_3_ matrices through the epoxide-assisted sol–gel process.

### 2.4. Transmission Electron Microscopy

**Figure 6:**
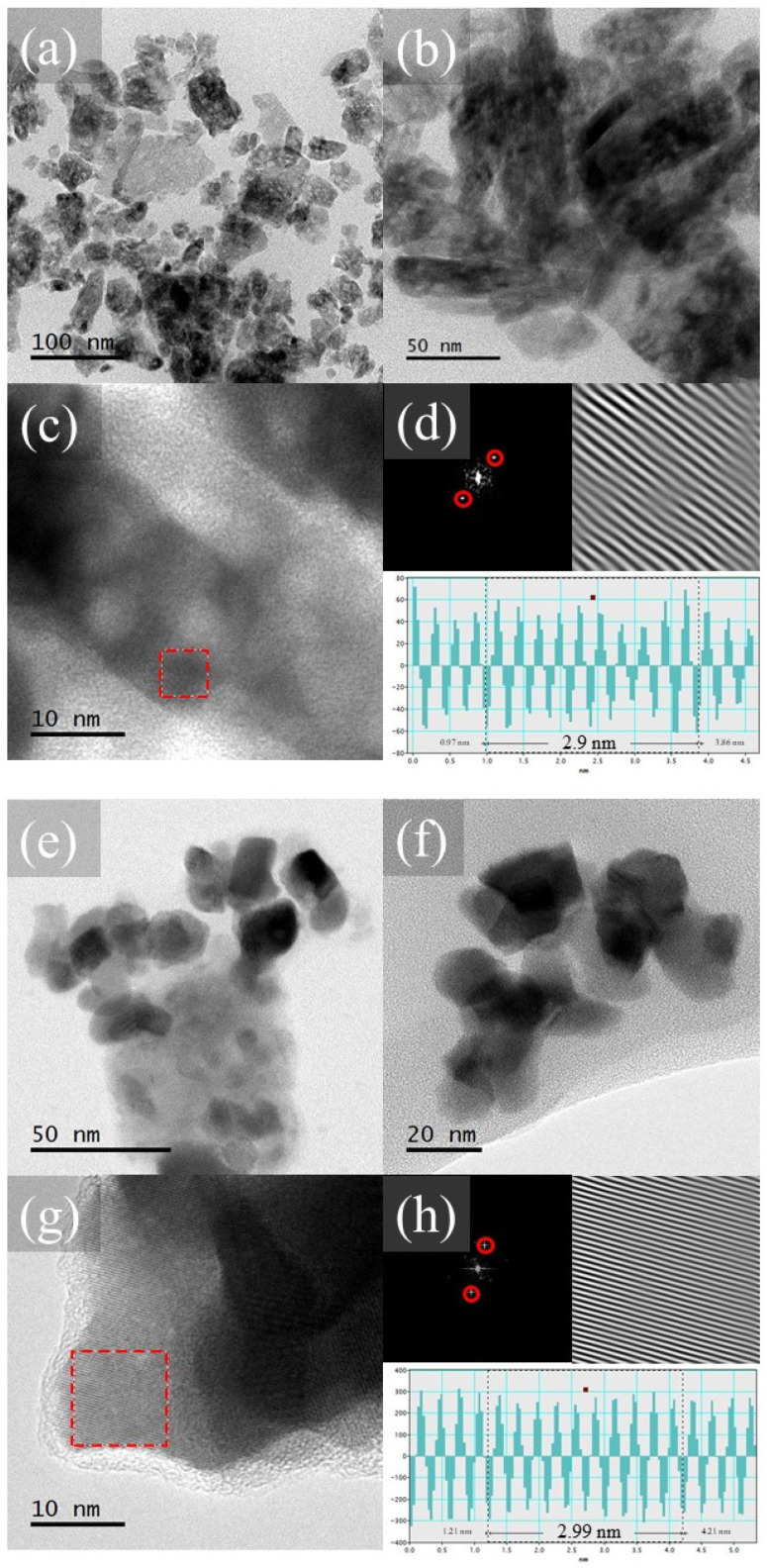
(**a**,**b**) TEM images of the CPLu10 sample at different magnifications, (**c**) HRTEM, and (**d**) SAED analysis; (**e**,**f**) TEM images of the CALu10 sample at different magnifications, (**g**) HRTEM, and (**h**) SAED analysis.

The transmission micrographs show similarities to the scanning micrographs presented above. Figure 6 depicts agglomerated particles in the CPLu10 sample (Figure 6a,b), while the particles of CALu10 (Figure 6e,f) are more dispersed. The planes visible in these samples were indexed through Fourier transform SAED analysis to determine the interplanar distances of the areas selected (see the red dotted squares in Figure 6c,g). These presented an average of 2.9 nm (Figure 6d) and 2.99 nm (Figure 6h) for a section of 10 planes corresponding to the CPLu10 and CALu10 samples, respectively.

This result coincides with the results obtained in the Rietveld analysis (see Table 2) and confirms what was found through XRD about the existence of the (222) plane and the formation of high-purity C-type crystalline structures without secondary phases. This all agrees with what is described in [4,49,51].

### 2.5. Photoluminescent Properties

**Figure 7:**
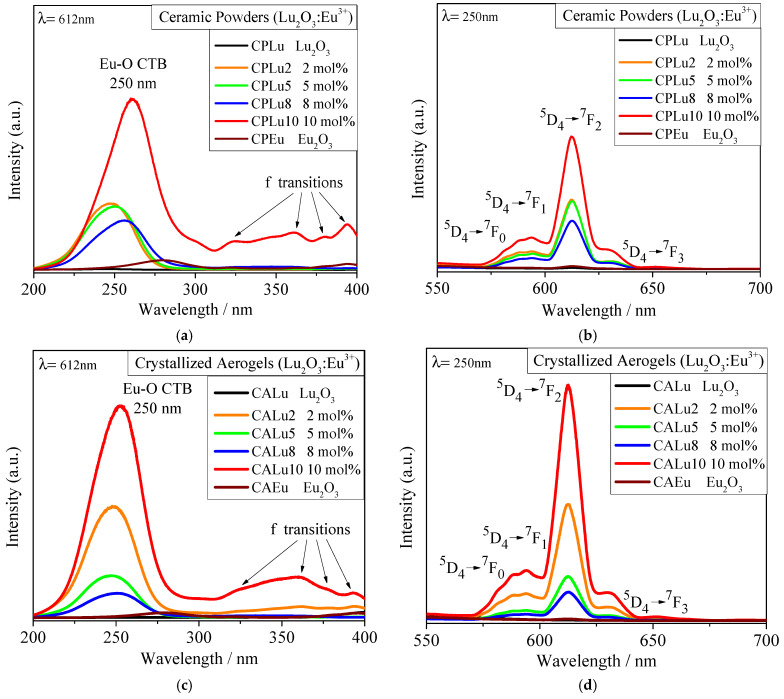
Photoluminescence study results: (**a**) excitation and (**b**) emissions of the ceramic powders, and (**c**) excitation and (**d**) emissions of the aerogels.

Figure 7 presents graphics of the CP (Figure 7a,b) and CA (Figure 7c,d) emission and excitation data. It is evident that the Lu_2_O_3_ matrix materials do not emit any type of signal on their own and that the photoluminescence is generated by the Eu^3+^ activator.

**Figure 8:**
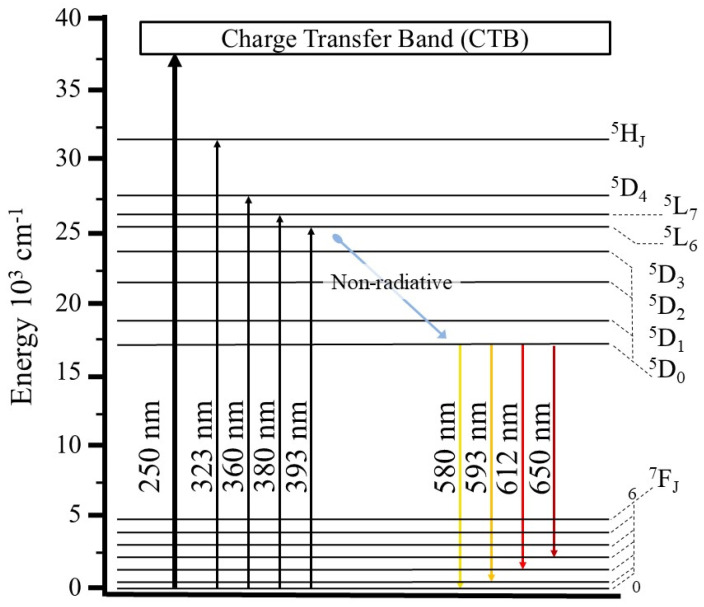
Energy-level diagram of the transfer energy of the Eu^3+^ in the CPs and CAs with the Lu_2_O_3_:Eu^3+^ system.

In the case of all the doped samples, a wavelength of 612 nm was able to excite the Eu^3+^ ions. While some slight signals corresponding to f transitions were present, it was the band around 250 nm corresponding to the charge transfer band (CTB) between Eu^3+^ and O^2−^ that showed the greatest intensity. Therefore, the emission spectra were obtained using an incident wavelength of 250 nm. In this case, bands associated with the energy transitions ^5^D_0_ → ^7^F_0_, ^5^D_0_ → ^7^F_1_, ^5^D_0_ → ^7^F_2_, and ^5^D_0_ → ^7^F_3_ of the Eu^3+^ ion appeared around 580, 590, 612, and 650 nm, respectively, although it should be noted that in all the photoluminescent samples, the hypersensitive transition of 612 nm predominated due to the proper energy transfer at the Eu(C_2_) → Eu(S_6_) sites. This process is described in [58] for the interpretation of europium (III) spectra; it is the same as that seen in other studies [4,44,45,50,51,59] and is schematized in the energy transfer diagram in Figure 8. However, in this case, in both materials (the CPs and CAs), the samples doped at 10 mol% exhibited the most powerful photoluminescence compared to the samples at other doping concentrations, and mainly with the samples in the Eu_2_O_3_ matrix. In addition, quenching did not occur in any of the samples, primarily because only low concentrations of the dopant were used [46].

**Figure 9:**
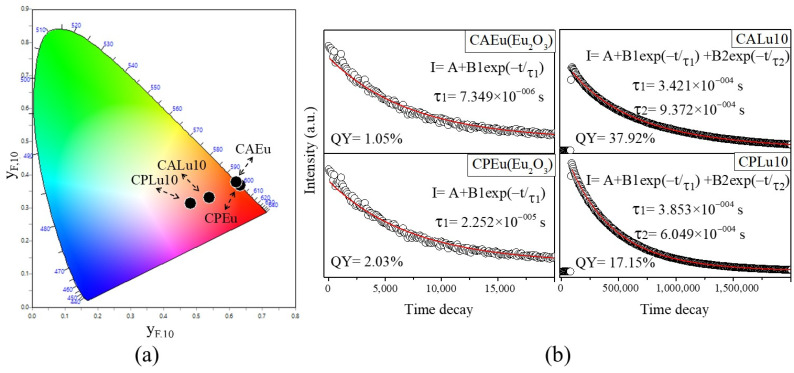
(**a**) CIE diagram and (**b**) results of the decay time and quantum yield of the Eu_2_O_3_ matrices and samples, showing strong photoluminescence in the CP and CA samples doped at 10 mol%.

The International Commission on Illumination (CIE, its acronym in French) chromaticity coordinates were calculated. In Figure 9a, the CIE locations of the Eu_2_O_3_ matrix samples (CPEu and CAEu) are plotted; these are located in the reddish-orange emission zone. The locations of the samples with powerful photoluminescence (CPLu10 and CALu10) underwent a change in emission color, tending more toward orange-pink. In Table 4, all the values obtained for the CPs and CAs samples are listed, together with the data obtained for the chromaticity coordinates for other red phosphors. As can be seen in Table 4, only the chromaticity coordinates of the Eu_2_O_3_ matrices are close to the values of both the coordinates for the commercial phosphor Y_2_O_2_S:Eu^3+^ (0.658, 0.340) and the NTSC-recommended standard (0.67, 0.33). All the values for the doped samples are far from the red emission region, suggesting that the interactions between the Lu_2_O_3_ and Eu^3+^ ions are characterized by a powerful and pure reddish-orange color.

As mentioned, it can be inferred that doping with Eu^3+^ was the main cause of photoluminescence because the quantum yield from the Eu_2_O_3_ matrices (Figure 9b) was very low at only 2% for CPLu and 1% for CALu. However, the samples doped at 10 mol% greatly exceeded these values, with figures of 17% for CPLu10 and 37% for CALu10. Furthermore, the decay curves are well-fitted into a single exponential for the CP samples and into a double exponential for the CA samples.

Furthermore, from the intensity of the emission spectra, it was possible to calculate the symmetry ratio (R) of the ^5^D_0_-^7^F_2_ electric dipole transitions relative to the ^5^D_0_-^7^F_1_ magnetic dipole transitions for both the CPs and CAs:

R = I(^5^D_0_ → ^7^F_2_)/I(^5^D_0_ → ^7^F_1_) (3)

The results are shown in Table 4 with the exception of the host matrices (CPLu and CALu), which did not present a luminescence response. The R values remained at around 4 for the doped samples, while for the CPEu and CAEu matrices, the values dropped to 2 and 1.45, respectively. This change in the asymmetric ratio suggests that the Eu^3+^ ions in the doped samples are localized in a non-symmetric environment in the crystal lattice, while in the Eu_2_O_3_ matrices, the Eu^3+^ is localized in a symmetric environment within the crystal lattice. It is important to note that R provides information about the symmetry environment of the crystallographic sites of the Eu^3+^ ions. Thus, if the value of R is high, Eu^3+^ tends to be located at asymmetric or non-centrosymmetric sites; if the value of R is low, Eu^3+^ tends to be located at high-symmetry sites or centrosymmetric sites. This could be why 10 mol% doping leads to better luminescent properties since, as seen in reports of other work [61,62], it is suggested that the greater the asymmetry of the Eu^3+^ ions, the stronger the Eu-O covalence, and hence, the greater and more powerful the emission intensity.

### 2.6. Analysis of Porosity by the Brunauer–Emmett–Teller (BET) Technique

**Figure 10:**
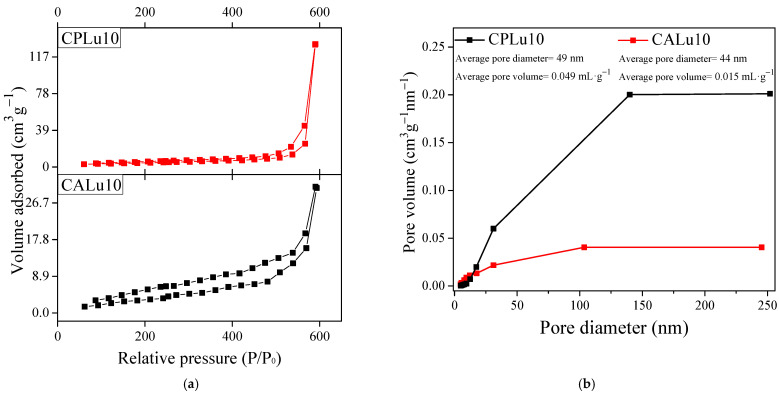
(**a**) N_2_ adsorption–desorption isotherms and (**b**) BJH pore-size distribution curves for the samples CPLu10 and CALu10.

The BET technique by nitrogen adsorption–desorption was used to calculate the pore volumes, the average pore diameters, and the specific surface areas of the CPLu10 and CALu10 samples. As established by Brunauer et al. [64], CPs exhibit a type-IV isotherm with an H4 hysteresis loop (Figure 10a). The surface area results were 5.36 and 5.17 m^2^·g^−1^ for CPLu10 and CALu10, respectively. The average pore diameter and volume values shown in Figure 10b for CPLu10 were 49 nm and 0.049 mL·g^−1^, respectively, while for CALu10, they were 44 nm and 0.015 mL·g^−1^.

The results suggest that crystallized aerogels have smaller diameters and pore volume sizes than ceramic powders. This could be due to the factors discussed in Section 2.3 on the results of the SEM analysis. In the case of the CPs, after heat treatment, a collapse occurred that caused particle agglomeration, which would mean a loss of porosity where the largest pores (macropores) persist. On the other hand, although the CAs also show a slight compaction from the heat treatment, they present a morphology with the shape of an interconnected three-dimensional structure as a result of the scCO_2_. The formation of this “open” structure must have resulted from the arrangement of the crystallites, and consequently, a greater number of micro- and mesopores were produced.

As explained by Rouquerol et al. [65], although the BET technique is widely used, it can produce errors when applied to microporous materials and should not really be used. Consequently, validly and reliably determining the porosity of aerogels is still considered a challenge, as they are synthetic materials with the highest porosity ever obtained. Because of this, it is likely that the adsorbate molecules underwent packing, which impeded gas permeability throughout the porous structure. Other factors that could affect the results are the type of pore and whether the material was adsorbent or not.

For everything described above, CALu10 was the most attractive and efficient sample, presenting some of the essential characteristics of a sensor, as mentioned in [1], such as high sensitivity, stability, good response times, and ease of synthesis. A good addition to this list would follow from testing whether these types of REO aerogels are non-toxic since, considering their nanometric properties, especially their porosity, the interaction of the surface area with the biological systems is still an area in which much exploration is needed. However, if the porosity of luminescent aerogels can be used to carry an active compound in a controlled manner, this class of materials could open the horizon for a new generation of products in nano biomedicine.

## 3. Conclusions

A novel series of crystalline materials consisting of a Lu_2_O_3_ system doped with Eu^3+^ and possessing photoluminescent properties were synthesized successfully by the epoxide-assisted sol–gel process. This method showed excellent results for the formation of stable gels, and subsequently, xerogels and aerogels, which can be crystallized so that materials of high purity and crystallinity are produced (in the form of ceramic powders and crystallized aerogels). Due to the similarity that exists between the CPs and CAs samples in IR spectroscopy, XRD, and EDS characterizations, it was determined that there are no chemical changes when modifying the molar% of Eu^3+^ and the difference is mainly reflected in the morphology due to the type of drying and the luminescent response of each system. Despite this, a correlation noted here that has not been reported in previous work concerns the size of the crystallites and how their formation was affected by increasing the concentration of Eu_2_O_3_ in the chemical composition of the materials. This also caused changes in the photoluminescence response, indicating that, in combination with the “open” and dispersed morphology presented by crystallized aerogels, it is possible to obtain a material with powerful and efficient photoluminescent properties, especially with doping at 10 mol%. Aerogels with photoluminescent properties have advantages over common biomarkers and drug carriers; therefore, some of the materials proposed in this project could have analytical applications in biomedical and related technologies; however, a barrier—or possibly a window of opportunity for researchers—can be noted in the lack of information relating to the toxicological damage that RE and aerogels can cause when they encounter biological systems.

## 4. Materials and Methods

To synthesize the gels, lutetium sesquioxide (Sigma-Aldrich, St. Louis, MA, USA, 99.9%) and europium sesquioxide (Sigma-Aldrich, St. Louis, MA, USA, 99.9%) were used. Hydrochloric acid (HCl, Fermont, México, 97%) was used to form the metallic salt. Ethanol (CH_3_CH_2_OH, J. T. Baker, BirthKansas City, MO, USA, 99.9%) served as the solvent, the gelation initiating agent was propylene oxide (CH_3_CHCH_2_O, Sigma-Aldrich, St. Louis, MA, USA, 99.9%), and the accelerator was citric acid monohydrate (C_6_H_8_O_7_•H_2_O, Sigma-Aldrich, St. Louis, MA, USA, 99.9%). Supercritical drying was carried out in a Quorum E3100 critical point dryer, and the CO_2_ used was acquired from the INFRA Group.

The following equipment and parameters were used to carry out the characterizations:

For IR spectroscopy, to determine the functional groups, a Perkin Elmer Spectrum 65 spectrometer was used, along with the potassium bromide (KBr) pellet technique. The transmittance was calculated for 2 min, covering wavelengths between 4000 and 400 cm^−1^.

X-ray diffraction was performed on a Bruker D8 ADVANCE diffractometer using the powder method. To establish the crystallographic-structural behavior, scanning was performed with Cu-Kα radiation covering the 2θ angle in a range of 20° to 80°. HighScore Plus software (v5.2) was used to conduct the Rietveld analysis and determine the cell parameters. The average size of the crystallites was obtained using the Debye–Scherrer equation.

Scanning electron microscopy was performed on selected samples of the crystallized aerogels, which were coated with gold using the sputter deposition technique for 5 s, with JEOL JSM-7800F equipment (Tokyo, Japan) at a voltage of 2 kV, and it was performed on selected ceramic powders with JEOL JSM-6701F equipment (Tokyo, Japan) at a voltage of 10 kV. To carry out the elemental chemical study by energy dispersive X-ray spectroscopy (EDS), these procedures took advantage of adapted detectors: an EBSD detector from EDAX for the crystallized aerogels and an INCAx-act detector from Oxford Instruments for the ceramic powders. Transmission electron microscopy (TEM) was performed with JEOL JEM-2100 equipment (Tokyo, Japan) under a bright field, and selected area electron diffraction (SAED) was performed using Gatan DigitalMicrograph software 3.01. 

For photoluminescence analysis, a Hitachi F-7000 (Tokyo, Japan) fluorescence spectrophotometer with a 150-W xenon lamp was used. The excitation and emission spectra were recorded based on the interaction ranges of the activator ion (Eu^3+^). These were produced using wavelengths of EX: 250 nm and EM: 612 nm. The analysis time for each result was 60 nm/min, using a voltage of 400 V and maintaining the slit opening at 10 nm. Color Calculator software (v2.0) was employed to calculate the values (x, y) of the chromaticity coordinates and establish the colorimetry (CIE). The time decay (λem = 611 nm) was calculated with an SC-15 device with a front phase sample holder for the powders and films, and the quantum yield was calculated with an SC-30 Sphere Integrator Module. Both techniques were performed on an Edinburgh Instruments FS5 spectrofluorometer position of Lu_2_O_3_ matrices through the epoxide-assisted sol–gel process.

Finally, the nitrogen adsorption–desorption analysis was performed using the BET technique in a Quantachrome AutosorbIQ unit, performing desorption for 18 h at 423 K in a 9 mm cell.

### 4.1. Synthesis

#### 4.1.1. The Sol–Gel Process

The precursors for the sol–gel process were in the form of chlorides (RECl_3_), which were obtained from a mixture of rare-earth sesquioxides (RE_2_O_3_) in 32.638 mmol of hydrochloric acid (HCl). Forming the host matrix of Lu_2_O_3_ and the matrix of Eu_2_O_3_ required only the mixture of 0.251 mmol and 0.284 mmol, respectively. Forming the different systems of doped samples, 2, 5, 8, and 10 mol% of Eu_2_O_3_ was mixed into the Lu_2_O_3_ host matrix. The reaction generated was as follows:

(4) $${\mathrm{RE}}_{2}O_{3}+6\mathrm{HCl}\to 2{\mathrm{RECl}}_{3}+3H_{2}O$$

Complete dispersion of RE_2_O_3_ (where for this project, RE can be Lu or Eu) in 6HCl was evidenced by the change in appearance from a whitish to a translucent solution, indicating the formation of the metal salt (2RECl_3_). Once the metal salt was formed, 34.782 mmol of CH_3_CH_2_OH was added as a solvent. At this point, the sol formed, and the hydrolysis and condensation reactions began. To promote condensation, propylene oxide (20 mmol) was added as an epoxy assistant [19,35]. This reaction was as follows:

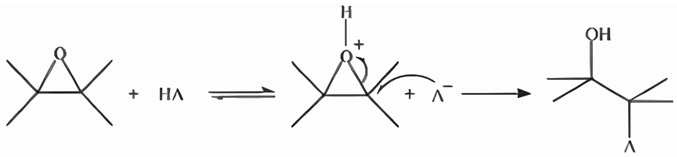
(5)

Citric acid monohydrate dissolved in ethanol in a 1:10 ratio was added immediately. This acted as a catalyst that caused the formation of a whitish mixture with a thick and lumpy consistency, identifiable as an alcogel. The entire process was carried out under constant stirring at a temperature of 353 K. The alcogel was then aged at room temperature until gravity stopped the flow of the viscous mixture, which occurred within approximately 24 h [8].

After this, wet gels were obtained that could be manipulated in subsequent processes. Although, at this point, the gels were in their most stable form, great care had to be taken when handling them so as not to collapse the microstructure because a homogeneous three-dimensional network confers adequate structural properties, such as mechanical strength, porosity, transparency, and a sizable specific surface area [66].

**Figure 11:**
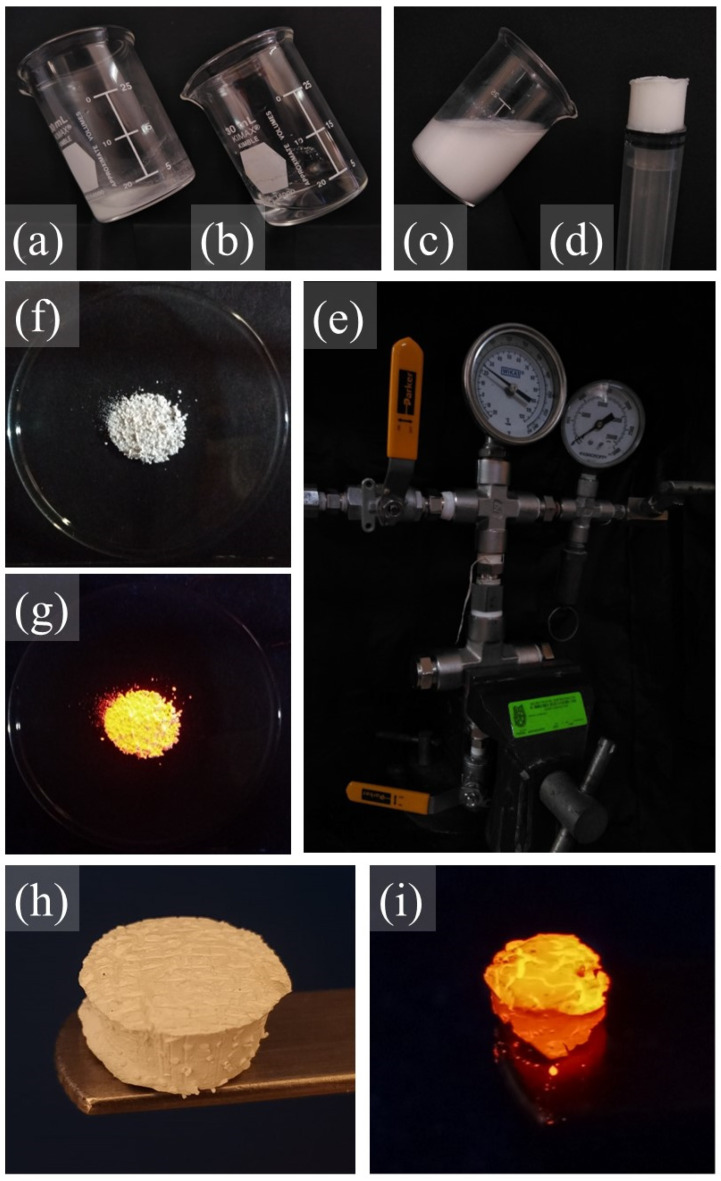
Stages in the sol–gel process used for the Lu_2_O_3_:Eu^3+^ system from metal salt to ceramic powders and crystallized aerogel: (**a**) metal salt, (**b**) sol, (**c**) wet gel, (**d**) aged monolithic gel, (**e**) general xerogel of the Lu_2_O_3_:Eu^3+^ system, (**f**) ceramic powders (the CPLu10 sample) under UV radiation, (**g**) supercritical drying chamber, (**h**) general aerogel of the Lu_2_O_3_:Eu^3+^ system, and (**i**) a crystallized aerogel (the CALu10 sample) under UV radiation.

Figure 11 shows the process, from the dispersion of the chloride salt (Figure 11a) and its change to a sol (Figure 11b), through its transformation into an alcogel (Figure 11c) and its transition to an aged monolithic wet gel (Figure 11d). At this stage, it is possible to obtain xerogels (Figure 11e), which, when thermally treated, are converted to ceramic powders (CPs) with photoluminescent properties (Figure 11f). Alternatively, by using a supercritical drying chamber (Figure 11g), it is possible to convert the aged gels into aerogels (Figure 11h), which when thermally treated become crystallized aerogels (CAs) that also have powerful photoluminescent properties (Figure 11i).

#### 4.1.2. Supercritical Drying of the Aerogels (scCO_2_)

Supercritical conditions were established for the CO_2_ to act as a supercritical fluid [18]. This occurred at a pressure of 73.7 bars and a temperature of 305.15 K. Once the aged gels were placed inside the supercritical drying chamber, the interior was filled with CO_2_. As the gas gradually entered the gel cavities and became a supercritical fluid, the liquid phase (CH_3_CH_2_OH) was displaced. The time required for complete drying was 3 days, with the CO_2_ being replenished every 24 h to ensure complete elimination of the liquid phase. As mentioned in [36], CH_3_CH_2_OH can be extracted with relative ease due to the high solubility of ethanol in supercritical CO_2_.

#### 4.1.3. Heat Treatment

Since materials obtained by the sol–gel process commonly occur in an amorphous form [67], both the xerogels and aerogels were subjected to thermal treatment to obtain the crystalline materials needed for the study: CPs and CAs. Heat treatment was performed at 1073 K for 2 h. This process facilitated the elimination of organic remains and solvent residues that remained anchored to the aged gels during the synthesis process and handling.

Figure 11e reveals that the xerogels did not undergo a significant change when converted into CPs (Figure 11f). While the aerogels (Figure 11h) suffered contraction when crystallized (Figure 11i), they were preserved in the form of monoliths, although they were very fragile to the touch.

## Figures and Tables

**Table 1:** Classification of the samples of ceramic powders and crystallized aerogels with the Lu_2_O_3_:Eu^3+^ system.

Ceramic Powder|Crystallized Aerogel	Eu_2_O_3_ mol%	System	Description
CPLu|CALu	0	Lu_2_O_3_	Host matrix
CPLu2|CALu2	2	Lu_2_O_3_:Eu^3+^	Doping
CPLu5|CALu5	5		
CPLu8|CALu8	8		
CPLu10|CALu10	10		
CPEu|CAEu	100	Eu_2_O_3_	Matrix

**Table 2:** Crystallographic data from the Debye–Scherrer equation and the Rietveld and SAED analyses.

Material	Sample	Crystallite Size Average (nm)	Rietveld Analysis D-Spacing (222) Plane (nm)	SAED Analysis D-Spacing (nm)
Ceramic Powders	CPLu	15.8	3	-
	CPLu2	13.11	3	-
	CPLu5	15.7	2.9	-
	CPLu8	9.6	3	-
	CPLu10	10.3	3	2.9
	CPEu	-	3.1	-
Crystallized Aerogels	CALu	17.7	2.99	-
	CALu2	10.4	2.99	-
	CALu5	17.1	2.99	-
	CALu8	8.2	3	-
	CALu10	13.5	2.99	2.99
	CAEu	-	3.1	-

**Table 3:** Lattice parameters and unit cell volumes obtained by Rietveld analysis for the CPs and CAs.

Sample		a/b/c [Å]	Volume [Å^3^]	α/β/γ [°]
Ceramic Powders	CPLu	10.38/10.38/10.38	1119.584	90/90/90
	CPLu2	10.40/10.40/10.40	1127.454	
	CPLu5	10.42/10.42/10.42	1133.297	
	CPLu8	10.45/10.45/10.45	1141.643	
	CPLu10	10.47/10.47/10.47	1148.084	
	CPEu	10.85/10.85/10.85	1280.276	
Crystallized Aerogels	CALu	10.39/10.39/10.39	1124.719	
	CALu2	10.41/10.41/10.41	1128.958	
	CALu5	10.41/10.41/10.41	1130.718	
	CALu8	10.45/10.45/10.45	1141.607	
	CALu10	10.45/10.45/10.45	1142.101	
	CAEu	10.87/10.87/10.87	1287.275	

**Table 4:** CIE chromaticity coordinates, relative luminescence intensity ratios (*R*s) of the (^5^D_0_-^7^F_2_)/(^5^D_0_-^7^F_1_) transitions, and quantum yields for the CPs, CAs, and other Eu^3+^-doped oxide compounds. “n.a.” (not applicable).

Sample		Chromaticity Coordinates (x, y)	R	Quantum Yield (%)	Ref.
Ceramic Powders	CPLu	-	-	-	n.a.
	CPLu2	(0.4983, 0.3388)	3.88	-	
	CPLu5	(0.5266, 0.3349)	4.1	-	
	CPLu8	(0.5128, 0.3243)	4.1	-	
	CPLu10	(0.4823, 0.3128)	4.2	17.1	
	CPEu	(0.6322, 0.3673)	2	2.03	
Crystallized Aerogels	CALu	-	-	-	
	CALu2	(0.4855, 0.3284)	4.1	-	
	CALu5	(0.4915, 0.3291)	4.1	-	
	CALu8	(0.4635, 0.3184)	4.09	-	
	CALu10	(0.5383, 0.3318)	4.6	37.92	
	CAEu	(0.6220, 0.3775)	1.45	1.05	
92% Gd-8% Eu		-	7.47	2.4	[60]
50ZnO:47B_2_O_3_: 3Nb_2_O_3_:0.5Eu_2_O_3_		(0.656, 0.343)	5.16	-	[61]
Glass-ceramics 25 h		(0.652, 0.348)	5.49	-	[62]
NTSC red phosphors standard		(0.67, 0.33)	-	-	[61]
Y_2_O_2_S:Eu^3+^		(0.658, 0.340)	-	-	[63]

## Data Availability

The data that support the findings of this study are available upon request from the corresponding author. The data are not publicly available due to privacy or ethical restrictions.
